# Supplementary material for: Are carbon emissions trading and green financial instruments synergistic? -Comprehensive quantitative research based on content analysis
Source: PLoS One. 2024 Mar 7;19(3):e0298601. doi: 10.1371/journal.pone.0298601 (PMC10919601; doi:10.1371/journal.pone.0298601)
Supplement: S1 Data — (ZIP) [file pone.0298601.s001.zip › Data/地级市Z_F工作报告.zip!/║┌┴·╜¡/║╫╕┌╩╨/└┤╫╘╣1⁄2╓┌║┼í╛┬φ┐╦╩2╛▌═°í┐_2017.docx]

各位代表：

现在，我代表市人民政府，向大会报告工作，请予审议。并请各位政协委员和其他列席人员提出意见。

一、过去五年工作回顾

过去的五年，是鹤岗发展历史上极不平凡的五年。面对经济下行压力持续加大的严峻形势，在省委、省政府和市委的坚强领导下，在市人大、市政协的监督与支持下，我们始终坚定发展信心，主动适应新常态，沉着应对困难和挑战，妥善处理矛盾和问题，经过全市上下的不懈努力，顶住了压力，经受住了考验，开创了经济企稳回升、民生持续改善、社会安定和谐、各项事业发展的良好局面。预计2016年主要经济指标，除规模以上工业增加值同比下降4%以外，其它指标均由负变正，实现了恢复性增长。地区生产总值实现272亿元，增长0.5%；固定资产投资95.9亿元，增长6.5%；公共财政收入17.8亿元，增长13.9%；社会消费品零售总额126.7亿元，增长9%；城镇居民人均可支配收入19741元，增长4.5%；农村居民人均可支配收入12882元，增长6%。虽然经济增长低于预期目标，但在困境中我们仍然实现了保工资、保运转、保民生、保重点，特别是体现经济发展质量和后劲的指标有所提升，为我市加速推进城市转型、同步建成小康社会夯实了基础、积蓄了动能。

——过去的五年，是经济结构不断调整、三次产业协调发展的五年。农村经济持续发展。粮食总产稳定在20亿斤以上。农业标准化生产面积发展到280万亩。农业产业化龙头企业、农民专业合作社、家庭农场和专业大户数量都有大幅度增加。实施了144项国家和省级重点水利工程。工业经济困中企稳。出台并落实支持工业企业发展若干政策措施，建立了企业服务中心和投诉中心。开展了“规上工业保卫战”，及时化解企业遇到的各类难题，确保存量增效、增量达效。第三产业稳中有升。现代物流商贸等新兴业态呈现良好发展态势。商贸流通业户数达到2.7万户，空车配货、仓储物流、信息服务、交通运输企业达到114户。旅游、金融和养老产业快速发展。累计接待游客和旅游收入分别增长16.5%和12.5%。金融机构存、贷款余额比2011年分别增长52.7%和96.1%。推进养老产业项目15个，促进了我市养老床位数量的增长和质量的提升。对外交往与合作不断扩大。积极推进对俄贸易，启动了入境落地签证和出境异地办照业务，口岸过货量逐年提高。

——过去的五年，是项目建设步伐加快、经济发展后劲增强的五年。累计实施千万元以上产业项目344个，完成投资328亿元。加快发展煤电化工产业。新建了中海油华鹤、征楠煤化工等一批重点项目，全市已经形成200万吨焦化、12万吨LNG、52万吨大颗粒尿素产能。加快发展石墨新材料产业。编制了产业发展规划，开展了探矿权、采矿权“两清、两整”行动，组建了鹤鸣云山矿业开发有限公司，引进了石墨烯粉体及下游石墨烯导电浆料、石墨烯润滑油、石墨烯涂料等新技术，奥星石墨、华升石墨烯、帝源石墨等项目相继建成。加快发展绿色食品产业。新建了万源稻米全产业链项目和一批稻米加工项目，全市形成530万吨的稻米加工能力，绥滨肉鹅基地、宝泉白羽鸡加工以及黑龙江网箱养鱼等特色项目实现产业化。招商引资取得突破。组织参加各类招商引资经贸洽谈活动50多项，引进项目728个、到位资金341亿元。狠抓了园区建设。市经济开发区入驻企业54户，并已晋升为省级工业园区。

——过去的五年，是民生保障力度加大、群众得到实惠较多的五年。尽管财政出现较大减收，但我们始终把民生支出放在突出位置，特别是向困难群众倾斜。全市民生支出年均增长12.5%。民生工程深入实施。就业创业形势总体向好，新增就业11.3万人，城镇登记失业率控制在4%以内。保障性住房建设实现突破，开工建设各类保障性住房6.2万套，改造农村泥草（危）房1.5万户。社保体系日趋健全，最低工资、养老金、城乡低保金等标准逐年提升，“五险”新增9.8万人。从2014年起救助龙煤鹤矿公司低收入困难矿工和支出型困难家庭1.7万人次。社会事业不断进步。各级各类教育均衡发展，标准化学校达到73所，特殊教育“三残”儿童入学率达到88%。医疗卫生服务功能日趋完善，市人民医院区域医疗中心、市妇幼保健院等投入使用，组建了5大区域医疗联合体，41项医改的目标任务全面完成。文化、体育、科技事业蓬勃发展，持续开展全市性大型群众文化活动，乡镇综合文化站、街道文化中心、村社区文化室、农家书屋实现全覆盖，市县公共图书馆、文化馆、博物馆、美术馆免费开放；广泛开展全民健身运动，加强群众身边体育设施建设，积极参加国内外各类重大赛事, 并取得了可喜的成绩；培育国家级高新技术企业8户、科技型中小企业346户和专利优势企业14户，建成了国家级石墨检测重点实验室和全国首家稻谷综合利用工程技术研究中心。社会形势持续稳定。大力推动安全责任体系建设，开展安全生产专项整治，各类生产安全事故起数下降38%；食品安全、物价稳控工作得力有效；加大信访矛盾纠纷排查化解力度，化解各类信访积案近3000件，圆满完成了重大敏感时期的信访稳定工作；加强社会治安综合治理，刑事、治安案件均呈逐年下降趋势，社会治安形势明显好转。

——过去的五年，是基础设施投入较多、城乡面貌发生变化的五年。重点实施了哈肇公路鹤岗至名山段、哈肇公路汤鹤界至鹤岗段等项目。新改扩建红旗路等城区主次干道46条；推进了红军路等300多条次道路步道板改造及维修；建设了育才路人行过街桥及运输路等跨河桥，改造了振兴桥等7座桥梁；新改扩建79条街巷道路。升级改造公园广场9个，新增绿地11.6万平方米。新建、改建路灯2670基。改造老旧供热管网61公里，全市集中供热能力达到2480万平方米。建设公交站廊101座和小型停车场4处。新建日处理垃圾950吨的生活垃圾处理场。拆除违法违章建筑1181户、4万平方米，整治“弃管楼”1486栋。全面完成了国家下达的减排指标，淘汰燃煤小锅炉159台、黄标车6043辆。中心城镇和美丽乡村建设扎实推进，5个乡镇晋升为国家级生态乡镇。

——过去的五年，是自身建设得到加强、政府执政能力提高的五年。制定了法治政府建设五年规划，主动接受市人大、市政协和人民群众监督，共办理人大代表建议363件、政协委员提案398件，办复率均达到100％。建立了政府权力清单、责任清单、负面清单和权力运行图。完成了新一轮政府机构改革。深化了投资类项目审批流程改革，推行了“一次告知、并联审批、一图审查”模式。开展了办公用房、公务用车、吃空饷等专项治理。加大审计监督力度，保障了各项重大政策措施有效落实。公共资源交易等全部面向社会公开。加强作风建设，严肃查处了一批违反中央八项规定的人和事。

此外，民族、宗教、外事、侨务、人防、司法、统计、地震、残联、老龄、档案等项事业都取得了新成绩。

各位代表，五年的变化令人振奋，发展成绩来之不易。这是省委、省政府和市委正确领导的结果，是市人大和市政协监督支持的结果，是全市上下拼搏实干的结果，凝聚了全市人民的辛勤汗水。在此，我代表鹤岗市人民政府，向全市人民，向各级人大代表、政协委员，向各民主党派、工商联、无党派人士和人民团体，向驻鹤的解放军、武警官兵和公安民警，向在鹤的中省直单位和所有关心支持鹤岗经济社会发展的各界人士，表示衷心的感谢!

在肯定成绩的同时，我们也要清醒地认识到，我市经济社会发展仍存在着一些亟待解决的问题：一是经济结构不尽合理，主要是工业结构单一，煤炭工业占规上工业比重为59%，“一煤独大”还没有从根本上改变；二是产业转型步伐不快，高技术含量、高附加值的产品和项目不多，发展接续替代产业和战略新兴产业的任务还相当艰巨；三是财税增收的压力较大，现有重点税源企业的增收空间有限，新上的税源项目大多处于建设期，一些已经竣工投产的项目还没有达产达效，难以对财税增长形成有力的拉动。四是民生改善任务艰巨，“三供两治”、空气质量、城市交通、公共服务等方面与群众要求仍有很大差距，还有不少历史欠账。五是社会不稳定因素增多，一些久拖不解的疑难纠纷以及因政策因素带来的矛盾和问题还很多，社会不稳定压力增大。六是安全生产形势严峻，安全生产基础薄弱、各级各部门监管力量不强、企业主体责任落实不到位等问题依然突出；七是政府自身建设仍需加强，少数干部精神不振、状态不佳，执行力和落实力还需进一步加强；政府职能转变滞后，经济发展环境还有待进一步优化。对于上述问题，我们必须敢于正视和面对，采取更加有力措施认真加以解决。

二、未来五年奋斗目标和2017年重点工作

各位代表，未来五年，是鹤岗推进结构转型、方式转变、动能转换的攻坚阶段，也是实施“十三五”规划、全面建成小康社会的关键时期。刚刚闭幕的市第十二次党代会，在总结过去五年成就、科学分析当前和今后一个时期形势的基础上，绘就了未来五年的宏伟蓝图。面对新形势、新任务、新要求，我们既要看到前进道路上的困难和挑战，更要看到潜在的机遇和优势，承担起历史赋予我们的责任。只要我们保持战略定力，坚定发展信心，进一步增强机遇意识、危机意识和赶超意识，不断激发内生动力，上下同心、埋头苦干，鹤岗就一定会在加速推进城市转型，同步建成小康社会，建设活力边城、幸福家园的征程中谱写出更加绚丽美好的新篇章！

未来五年，政府工作的指导思想是：高举中国特色社会主义伟大旗帜，深入贯彻习近平总书记系列重要讲话精神，特别是持续贯彻落实对我省两次重要讲话精神，统筹推进“五位一体”总体布局和协调推进“四个全面”战略布局，牢固树立新发展理念，坚持稳中求进工作总基调，深化供给侧结构性改革，扎实做好“三篇大文章”，创新实施“五大规划”，深入推进“龙江丝路带”建设，实施“生态立市、产业强市、开放活市、人才兴市”四大战略，培育和发展“绿色矿业、生态农业、文化旅游、外贸物流、战略新兴”五大产业，着力推进结构调整，着力推进创新创业，着力推进民生改善，着力推进重大基础设施建设，着力推进城市功能提升，着力推进社会治理，着力推进政府自身建设，打造“龙江东部工业强市、中国北方鱼米之乡、中俄界江旅游胜地”，为加速推进城市转型，同步建成小康社会，建设活力边城、幸福家园而努力奋斗！

按照市第十二次党代会的部署要求，未来五年，我市经济社会发展的预期目标是：地区生产总值年均增长13%左右；规模以上工业增加值年均增长12%左右；固定资产投资年均增长15%左右；公共财政收入年均增长17%左右；社会消费品零售总额年均增长8%左右；外贸进出口总值年均增长10%左右；城镇居民人均可支配收入年均增长6.5%以上；农村居民人均可支配收入年均增长7%以上。

各位代表！实现加速推进城市转型，同步建成小康社会，建设活力边城、幸福家园的宏伟蓝图，必须从完成今年的目标开始。2017年，是实施“十三五”规划的重要一年，是供给侧结构性改革的深化之年，也是新一届政府工作开局之年，更是全市各项工作“打基础、求突破”的关键一年，做好今年的工作意义重大，任务艰巨。

2017年，我市经济还处于底部运行期，发展还面临诸多不确定因素，为确保市第十二次党代会确定的目标如期实现，我们必须勇挑重担，强力攻坚。经济社会发展的主要预期目标是：地区生产总值力争实现两位数增长；规上工业增加值增长10%左右；固定资产投资增长10%左右；公共财政收入增长6%左右；社会消费品零售总额增长9%左右；外贸进出口总值增长10%左右；城镇居民人均可支配收入增长6.5%以上；农村居民人均可支配收入增长7%以上。

（一）坚持抓产业、兴业态，打牢转型发展的基础

要认真贯彻落实习近平总书记关于“五个要发展”、“做好三篇大文章”及“抓好五头五尾”的重要指示，实施“产业强市”战略，以提高发展质量和效益为中心，注重市场导向、动能转换、路径拓展和模式创新，大力发展“五大产业”，切实把产业项目建设、创新驱动、新增长领域培育结合起来，在具有鲜明供给优势的领域持续发力，把优势资源变成产品和服务，提高实体经济的竞争力。

增强工业经济实力。强化工业的主体地位，坚持激活存量与引进增量并举，扩张总量与提升质量并重，努力打造“龙江东部工业强市”。一是向激活现有存量和改造提升传统产业要增量。积极运用先进适用技术提升传统产业，运用信息化、绿色化、服务化改造传统产业，运用创新商业模式助推传统产业，促进煤炭、食品、饮料、建材等产业向中高端迈进，把旧的存量转化为新的增量。要抓好现有企业的运行，稳住存量，特别是要稳定煤炭生产，用安全保生产、保效益，为转型积累财富、赢得时间。要改造提升“百年龙江酒业”等传统企业，把“老字号”品牌做大做强、做优做久。二是向资源开发和精深加工 要增量。依托域内资源，推动资源型产业链条向下游延伸，提高资源精深加工比重，增强市场竞争力。围绕发展绿色矿业，推进煤电化工产业。以“煤头电尾”、“煤头化尾”为抓手，通过“稳煤上醇”、“稳肥增品”、“稳焦扩气”等延伸产业链条，全力推进中海油华鹤10万吨乙二醇、50万吨复合肥和50万吨BB肥及征楠焦粒制气等项目，加快打造“新型精细煤化工生产基地”和“煤制氮肥、高效肥生产示范基地”。加大对腐殖酸、大理岩、硅石等矿产资源的开发利用，促进优势资源向优势企业、深加工企业配置，努力把资源优势转化为产业优势。三是向发展战略性新兴产业和科技成果产业化要增量。加快发展新材料、生物与新医药、新能源等产业，培育增长新动力。新材料产业，突出石墨综合开发利用，加快建立石墨新材料研发中心，谋划建设大型石墨基础材料产业基地和中高端产品基地，重点打造新能源材料、密封散热材料、冶金耐火材料、超硬材料和石墨烯等产业链，推进华升石墨烯润滑油、奥星石墨二期、国信通动力电池和智能手机等项目。突出陶粒页岩综合开发利用，重点扶持惠鹏超轻页岩陶粒、上德轨道交通吸音板等项目。生物与新医药产业，突出新药研发、中药和中成药的二次开发，重点扶持三精千鹤、未名和双兰星等制药企业，力争用2至3年时间，实现医药产值倍增。新能源产业，突出自然资源、粮食深加工废弃物资源和工业余热余压资源利用，重点推进大唐风电二期、吉阳光伏发电等项目。

推动农村经济加快发展。坚持把推进农业供给侧结构性改革作为农业农村工作的主线，培育农业农村发展新动能，大力发展生态农业，发挥农业生态系统的整体功能，推进农林牧渔各业协调发展，努力打造“中国北方鱼米之乡”。一是调好调顺调优农业结构。以市场为导向，调整种植业结构，重点扩大蔬菜、鲜食玉米、经济作物的种植规模。大力推进“两牛一猪一禽”规模化、标准化养殖。积极推广网箱养鱼、稻田养鱼、稻田养蛙、稻田养鸭等生态养殖模式。加快发展林菌林果、林下种植、林中养殖、林中药材等林业经济。二是增加绿色优质农产品供给。严格执行农业“三减”行动计划，强化绿色、无公害食品基地建设，重点在做大规模、质量安全监管上下功夫，严格落实重大动物疫病防控措施，让老百姓吃上放心米、放心菜、放心肉。三是推进农业产业化，促进农村一二三产融合发展。加强与中粮集团的战略合作，推动合作项目加快实施，积极培育农业产业化龙头企业，以“粮头食尾”、“农头工尾”为抓手，打造水稻、大豆、玉米、畜禽、蔬菜、山特产品精深加工和酒类制造等产业链，推进万源米糠油二期、兴汇玉米深加工、经纬豆制品加工、新北国啤酒二期、金鹤啤酒及功能饮料等项目。要全面强化营销，实施创牌战略，使企业和农民在价值链上获得更多收益。

加快发展现代服务业。把发展现代服务业作为推动经济转型的重要力量，注重产业融合发展，推动生产性服务业向专业化和价值链高端延伸、生活性服务业向精细化和高品质转变，培育新的经济增长点、市场消费点和就业拉动点。一是推进文化旅游产业快速发展。以“龙江三峡”为龙头，突出抓好“一江两岛三峡”工程，叫响“游龙江三峡、赏两国风光”旅游品牌，努力打造“中俄界江旅游胜地”。挖掘利用文化资源，打造具有鹤岗特色的矿山、抗联、垦荒等文化旅游产品。全面提升赏冰乐雪景区的规划设计和建设水平，打造冰雪文化、旅游和体育跨界融合的旅游新亮点。规划建设“兴山要塞”红色探险旅游项目，提升松鹤西湖、鹤立湖、清源湖景区建设水平，打造各具特点的“三湖”景区。大力发展森林度假游，全面推行林区公园、景点免费开放。举办中俄界江文化旅游活动，推动我市与俄罗斯犹太州旅游深度合作发展。设立旅游产业专项基金，加大策划宣传推介力度，提升我市旅游业的知名度和影响力。二是大力发展外贸物流产业。实施“开放活市”战略，促进对外贸易向全方位、多层次、宽领域发展。今年，要完善中俄边民互市贸易区功能，推进综合保税区建设。大力培育外贸经营主体，做实做强进出口加工基地，加大对俄矿产、木材等资源的开发力度，支持赴俄农业种植企业扩大规模、多元发展。推进我市农机、商贸、豆制品、肉制品等企业与俄方对接，达成新的合作项目。加快发展现代物流业，建设由名山口岸通往俄罗斯的物流通道，以南翔国际物流园区为龙头，推进现代物流产业园区建设，加快形成集运输、仓储、配送、物流加工、物流金融等业态于一体的产业集群。三是加快发展养老健康产业。积极开发“南病北治”产品，谋划建设“南病北治、北药南用”旅游健康养老示范基地。开发鹤岗道地药材，推出中医“治未病”产品和服务。发展“医养康”新模式，重点推进市中医院“医养康”综合体二期等项目。四是进一步发展现代商贸流通产业。扶持传统商贸企业转型升级，引进集购物、休闲娱乐、旅游文化、餐饮服务等功能于一体的城市综合体和商贸中心，重点推进新凯龙城市综合体、宏益家电形象体验店和金鹤啤酒广场花园等项目。加快推进大型农副产品市场建设。加快发展电子商务，成立电商工作机构，设立电商产业发展基金，建设电商产业园区，引进和培育电商企业，推进跨境电商项目建设。支持六公里超市、惠众电子商城等企业做大规模。五是着力发展文化产业。建立文化产业项目库，引进和培育各类传媒、出版、广播电视、文化创意等企业。筹建鹤岗艺术品网上交易平台。发展IT动漫产业，重点推进漫狐动漫网络游戏等项目。发展演艺娱乐业，支持和引导各类艺术表演团体，结合我市特有文化，进行艺术创作、生产和营销。六是积极发展体育产业。巩固体育彩票在体育产业中的特殊地位，加快水上健身中心、松鹤滑雪场建设。丰富体育服务产品供给，积极引进和办好各类高水平赛事，引导体育消费。七是发展信息服务业。继续推动基站和数据中心等基础设施建设，实现光纤宽带和4G网络深度覆盖。加快开发系统软件、管理软件、网络安全软件和数据库，重点推进尚华大数据、优易云天云基地等项目。

（二）坚持抓基本、守底线，打牢民生改善的基础

经济困难时期，更要关注群众的基本生活。要坚持可用财力向民生投入，把群众最需要、最关心、最期盼的事情办实、办好。

稳定扩大就业。采取积极的就业扶持政策，以创业带动就业，完善就业服务体系，加强技能培训，多渠道开发就业岗位，促进煤矿下岗职工、高校毕业生、贫困人口等重点群体有效就业，城镇登记失业率控制在4%以内，让城镇“零就业”家庭至少有一人就业。着力改善人民群众居住条件。用好用足上级政策，加大主城区棚户区改造力度，重点打好兴山、工农棚改攻坚战。对主城区老旧小区要进行修补，搞好绿化美化，完善必备的功能设施，改善居住环境；对新建棚改小区要确保设施健全、功能完善，提高生活便利性，让老百姓居住有好房子、出行有好环境。健全社会保障体系。以保基本、兜底线、促公平、可持续为准则，持续扩大“五险”覆盖面，提高保障层次和水平，实现应保尽保。健全社会救助体系，建立“救急难”制度，提高城乡低保补助标准，达到人均570元/月，做好重大传统节日期间各类困难群众的救助，抓好特殊困难群体的精准救助，实现应救尽救。建设殡葬服务设施，提高殡葬服务业水平。提升社会养老服务质量。办好公办保障性、民办公益性养老福利机构，重点抓好失能半失能护养院、兰天养老公寓等项目。启动居家养老服务试点工作，探索“互联网+养老”模式，为老年人提供方便、经济、快捷、高效的居家养老服务。促进教育均衡发展。优化学校布局，改善办学条件，推进各类教育协调发展。今年，要创建多所省级标准化示范幼儿园。积极帮助鹤岗师专解决发展难题，整合职教资源，推动职教与产业深度融合，构建以就业为导向的现代职业教育体系。扩大特殊教育办学规模，建成自闭症儿童特教中心。加快学校信息化建设步伐，全面完成“三通两平台”建设任务。加强中小学校园文化、体育建设，创建一批文化、体育特色学校。深入开展乱补课乱办班专项整治，严查重处败坏师德师风行为。完善助学体系，资助家庭困难学生，让每一个孩子都能上得起学、上得好学。提高人民群众健康水平。继续深入开展“卫生惠民工程”，执行医疗减免救助政策，启动城市公立医院综合改革，做实12类45项基本公共卫生服务项目，支持社会资本兴办医疗机构，继续推进分级诊疗、双向转诊等制度建设，推进医生多点执业，让群众看得上病、看得起病、看得好病。推动城市文化提质升级。文化是城市的气血，气血充足城市才有活力。要加强城市历史建筑及具有历史代表性文物的挖掘、保护和利用，丰富城市文化内涵。要保障文化事业必需的资金投入，建设必备的文化场馆。今年，要建设多功能档案馆，增加群众艺术馆活动场所，修缮人民剧场，完成博物馆布展，建成100个“互联网+专网”数字图书馆终端，推进街道、社区、乡村文化场所建设。支持引导群众开展身边的文化活动，支持各类民间文化协会的发展。开展文化名人家乡行活动，举办鹤岗开发建设100周年系列活动，带动文化热，提升文化品位。加快发展体育事业。体育要贴近群众，融入百姓生活，要举办广大群众喜闻乐见、易于参加的各类体育赛事，继续开展“百万青少年上冰雪”和“赏冰乐雪”系列活动。支持开办各类服务群众的体育技能培训，让老百姓想玩、会玩、玩得好。改造老旧体育场馆，建设群众身边的体育场地设施。抓好社区建设。创建标准化社区，提高社区工作人员待遇标准，改善社区办公场所条件，积极探索“居民＋业主委员会＋社区”的管理、服务新模式，让群众诉求有人听、事有人管、难有人解。加强物业管理。今年，将重点改造中心区“弃管楼”和龙煤鹤矿公司移交的住宅楼，解决小区环境脏乱差、物业管理水平低的问题，加强标准化小区建设，让小区绿起来、亮起来、美起来。扎实推进脱贫攻坚。坚持精准扶贫、精准脱贫，加大产业、教育、健康扶贫力度，决不让一个村屯、一个群众在全面建成小康社会的进程中掉队。同时，要下力气抓好城区贫困人口脱贫工作。

（三）坚持抓谋划、快推进，打牢重大基础设施建设的基础

坚持把重大基础设施建设作为推进供给侧结构性改革补短板的重要内容，切实抓好人民群众期盼的高铁、机场、公路等重大建设项目，持续用力，务求实效。铁路建设，做好鹤佳铁路既有线路改造、鹤名铁路等项目前期工作，积极谋划鹤佳高铁项目。机场建设，做好鹤岗支线机场前期工作，务求取得实质性进展，确保2020年前建成投用。公路建设，开工建设绥名公路、城市环路，启动市区至红旗林场等旅游公路工程。谋划鹤伊高速公路项目。口岸建设，加快萝北名山港码头改扩建，启动绥滨中兴港建设工程。水利建设，开工建设关门嘴子水库，确保2020年成功蓄水。推进细鳞河水库除险加固工程。“三供两治”工程，新建饮水改造提升工程5处；改造城市供热老旧管网，彻底解决年久失修、管网热损失和跑冒滴漏问题。推进2×35万热电联产项目；启动静脉产业园一期工程，推进垃圾焚烧发电项目。谋划建设海绵城市。

（四）坚持抓完善、强功能，打牢城市建设的基础

坚持提升老城区、完善新城区，实施生态修复、城市修补工程，增强城市对经济发展和人民生活的综合承载能力。

启动生态修复工程。实施“生态立市”战略，坚持绿色发展理念，严格落实环保法律法规，加快推进生态保护和修复，全力打造低碳城市。一是着力推进绿色发展。严格项目环保准入条件，特别是对新建煤化工企业，要执行最严格的大气污染物和污水排放标准，引导企业转变高耗能、高排放、高污染的发展模式。建立健全矿山生态环境和地质灾害监测体系，抓好煤矿边坡治理、尾矿治理、采空区治理、土壤改良、植物修复和水资源修复，防止新增污染和破坏。开展废弃矿山治理，重点对岭北露天矿进行生态修复和综合利用，打造“绿色矿山”。二是着力强化污染防治。推进大气污染防治，抓好工业废气治理、清洁生产等重点节能减排工程。加快10蒸吨以下燃煤小锅炉撤并和“黄标车”淘汰；推进水污染防治，实行“河长制”河湖管理模式，加大黑臭水体治理力度，提升城区沟河水质；推进土壤污染防治，开展污染治理与修复，改善土壤环境质量。实施建设用地准入管理，防范人居环境风险。三是着力加快生态创建。加强林区的规划、建设、管护工作，提高管理层级，防止林区乱批乱建、乱砍乱伐，保护好每一棵树、每一片草、每一块湿地、每一条溪流。坚持城区林区协同发展，统筹实施城区绿化、林区造林和义务植树工程，加强采煤沉陷区和棚户区腾空土地的治理、收储和复绿，增加区域性综合公园数量和绿地面积，加强森林自然保护区和森林公园建设，积极创建国家生态城市、森林城市。今年，将完善城区行道树布局，推进红旗路、跃进路等路段的绿化改造；启动建设城市北郊森林公园和南山路景观公园。

启动城市修补工程。坚持以高标准规划为引领，以完善城市功能为重点，以加强城市管理为突破口，努力打造和谐宜居、富有活力的现代化城市。一是坚持高起点规划城市。推进“多规合一”，树立“规划即法”理念，强化规划执法，坚决不允许再发生私批乱建、未批先建、批少建多等现象，切实维护城市规划的统一性和权威性。二是坚持高质量建设城市。解决“断头路”、“瓶颈路”和“大货车进城”等问题，重点建设国防路、南岗路、南山路、红旗路等城区骨干道路，规划建设南岗路跨国铁桥梁，打通城市南部路网交通瓶颈，让城区交通更顺畅。解决背街背巷、居民小区夜间无亮化等问题，实施12条道路老旧路灯及50栋重要楼体亮化改造工程。解决城区“停车难”的问题，充分利用路边、人行道及周边可用空间，建设一批小型停车场和停车位。三是坚持精细化管理城市。加强环境卫生治理，重点开展建筑工地扬尘和车辆扬尘治理，规范露天市场秩序，查处私搭乱建行为。加强建筑市场治理，大力整顿工程建设不规范和违反法定建设程序等问题。加强交通秩序治理，常态化整治“黑车”顽疾。加强超限超载治理，将治理超限超载工作纳入全年相关单位考核指标，对不作为的责任单位一票否决。开展文明城市创建活动，全面提高市民文明素养，进一步营造干净、整洁、文明、有序的城市环境。

（五）坚持抓安全、保稳定，打牢社会治理的基础

推进社会治理，关乎经济社会发展，关乎社会和谐稳定，关乎人民群众切身利益，必须持之以恒、长抓不懈。

强化安全生产工作。严格落实安全生产责任，抓好煤矿、道路交通、消防、非煤矿山、危险化学品、食品药品、环境保护、重大建设项目及建筑施工等重点领域和车站、商场、学校等人员密集场所的安全生产。重点加强煤矿安全监管，严厉打击违法违规开采行为。违法违规开采就是谋财害命，就是破坏经济发展和社会和谐，必须严厉打击，毫不手软。坚决用铁的手腕抓安全，用铁的规章促安全，用铁的纪律管安全，最大限度地减少安全生产事故的发生。

强化群众诉求办理工作。打造“阳光信访”，推行信访事项受理、办理、督办全过程公开，缩短办理周期，提高工作效率。打造“责任信访”，严格执行信访接待日、首问负责和领导包案等信访工作制度，积极化解信访积案，切实解决好群众反映强烈的问题。打造“法治信访”，进一步畅通群众诉求渠道，严格按照法定途径处理信访投诉请求事项，确保群众提出的问题依法依规得到解决。依法严肃处理无理缠访、闹访等违法行为。

强化社会治安综合治理工作。深化“平安鹤岗”建设，抓实公安“六大工程”，加快完善立体化、信息化社会治安防控体系。开展严打整治专项行动，坚持“四侦同步上案”机制，重点打击命案、严重暴力犯罪、多发性侵财犯罪、新型犯罪和网络犯罪等违法犯罪行为。加大显性用警力度，提高见警率和管事率，进一步提升群众的安全感。强化虚拟社会管理，健全网络舆论引导机制。加强政府应急指挥平台建设，完善各类预案，提高应急处置能力。

三、做好2017年工作的保障措施

一是抢抓重大政策机遇。要紧紧围绕“五大规划”、“新一轮东北振兴战略”、“东北振兴三年滚动计划”和“建设石墨新材料产业集群”等政策的投资方向，抓紧储备一批事关长远发展、民生福祉的重大项目。要把握政策导向，认真谋划论证，切实做好项目可研、初设等前期工作，积极主动与上级部门对接，尽最大可能争取更多的项目列入国家和省投资计划，最大限度承接政策红利。

二是加快产业项目建设。产业项目建设是拉动经济增长的重要引擎，是市委确定的“一号工程”，必须举全市之力，予以攻坚。要解决推进不力的问题，建立领导包保推进新机制。要给各县区压担子，深入开展产业项目“四比四看”活动。要储备一批，谋划储备一批投资规模大、科技含量高、产业关联度强、惠民效益好的优质项目。要落地一批，对已建立联系的项目，要密切跟踪，紧盯不放，促进项目早日签约落地。要开工一批，做好项目的立项、选址、用地、环评、能评、安评等前期工作，确保早日开工，提高产业项目的生成率。要投产一批，帮助项目解决在原料、资金、要素、运输等方面存在的问题，保证按时投产，尽快达效。

三是大力推进投资兴业。要向引入外来资本要发展，围绕我市的“五大产业”，大力开展以商招商、专业招商、产业链招商。招商要轻形式、重实质，在招外商外资的同时，高度重视内商内资，引导鹤岗籍在外成功人士回乡投资创业，全力支持本地企业家投资兴业。要营造亲商安商富商环境，让企业家在鹤岗受尊重、有地位、有钱赚，把根留在鹤岗。要组建鹤岗市投资合作机构，完善激励机制，重奖投资兴业有功人员，充分调动全社会投资兴业的积极性。要解决我市民营经济偏弱的问题，大胆解放思想，落实支持政策，放手发展民营经济，进一步激发民营经济活力和创造力，充分释放民间投资潜力，激活民间创业热情，培育更多的像喜家德、比优特一样的创业者。要建立企业家联谊会、咨询委员会和各类行业协会商会，使之成为服务企业发展、促进投资兴业的重要平台。

四是推动全民创新创业。解决社会创新创业活力不足的问题，要打造“双创”平台，加大政府创业资金投入力度，市、县区两级都要成立“双创”中心，加快创业孵化基地、企业创新基地、科技企业孵化器、“互联网+”应用平台等创新创业平台建设，引导各类新兴业态入驻平台。要培育“双创”主体，突出抓好青年创业，持续推进科技人员、大学生、农民、城镇转移就业职工4支队伍创新创业，使之逐步成为支撑鹤岗发展的重要力量。要鼓励企业与高校、科研院所开展协同创新，组建产业技术创新战略联盟。要培养“双创”人才，实施“人才兴市”战略，建立健全人才培养选拔、评价使用、流动配置机制，坚持引进和培养并重，加快人才队伍建设。

五是持续优化发展环境。要解决我市发展环境不优的问题，必须处理好管理与服务的关系，不能“一管就死、一放就乱”，进一步精简办事程序，缩短项目运作周期，降低投资者的营商成本，营造良好的政策环境、服务环境、法治环境、市场环境、社会环境和人文环境。要坚持整治重心下移，聚焦企业和群众反映最强烈的问题，紧盯关键岗位、重点部门、涉企行业和服务窗口单位，对破坏发展环境的行为要立场鲜明，典型查处，决不姑息。要解决园区散、小、乱和功能不全的问题，统筹抓好园区规划、建设、管理和运营，完善市经开区职能，推进基础设施建设，提升园区承载力和竞争力。要解决各区之间在项目选址、税收征缴等方面的问题，发展飞地经济，推动资源共享、土地共享、税收共享，促进区域协调发展。

六是全面深化各项改革。深化审批制度改革，继续做好行政审批事项“接放管”，建立部门权力清单、责任清单动态调整机制。今年，要选择一个区作为探索实施负面清单管理模式的试点。要深化商事制度改革，推进企业登记便利化，实施“多证合一”企业登记制度，强化事中事后监管，推动“双随机、一公开”监管方式全覆盖。要深化公共资源市场化改革，对集中供热热源、供气、供水、污水垃圾处理、医疗、教育、城市基础设施、公共网络、公共交通等经营性公共资源，都要采取招投标的方式进行公开配置。要深化售电侧改革，抓好电改试点工作，组建拥有配电网的售电公司，进一步降低企业用电成本，打造“电价洼地”。要深化事业单位改革，从控编减编做起，解决人力资源配置失衡、财政供养负担过重等问题。要深化就业、社保、教育、医药卫生、文化、体育等民生领域改革，处理好公平和效率的关系，为改善民生提供制度保障。

七是强化政策引导力度。今年，将设立产业发展基金、青年创业基金、中小微企业担保基金和助保金，充分发挥政府资金引导和放大作用，扶持产业和企业加快发展。要支持发展融资性担保机构、小额贷款公司，积极组建农商银行、民间借贷登记中心。要引导企业树立资本经营意识，运用联合发行债券、私募基金、资产证券化等融资工具加快发展。支持帮助具备条件的企业上市。要构建多元化投融资体系，推进政府与社会资本合作PPP项目建设。要加强税收征管，既要培育税收贡献较大的财源项目，又要抓好组织收入，确保应收尽收，为经济发展、民生改善提供财力保障。

八是广泛开展区域合作。解决我市域内合作不够深入、域外合作不够紧密的问题，要继续支持龙煤鹤矿公司改革发展，推进市矿在发展规划、矿产资源精深加工、生产要素配置、城市建设、群众生产生活等方面实现深度融合。特别是要妥善抓好人员分流、“两供一业”移交、职工救助等项工作，帮助企业摆脱困境。要加强与农垦宝泉岭分局、鹤北林业局的合作，在推进生态农业、绿色食品、文化旅游产业和小城镇建设等方面形成合力，携手推动区域发展。积极推动华能鹤电公司、中海油华鹤公司利用人才、技术、装备和管理的优势，抓好产业链条的延伸，全力以赴助其发展壮大。要深度融入东北东部（12+2）区域合作，重点与伊春、佳木斯开展合作，共同推动文化旅游、健康养老产业转型发展。要紧紧抓住我省与广东省对口合作的机遇，加大对接力度，努力在粮食产销、绿色食品加工、文化旅游、健康养老等重点领域合作上取得突破性进展。

四、以改革创新精神加强政府自身建设

要全面深入持续贯彻习近平总书记系列重要讲话精神，坚持目标导向与问题导向相结合，坚持底线思维与法治思维相统一，把“认真、实干、创新”精神转化为具体行动，勇于担责，奋力前行，努力建设人民信任和满意的政府。一是增强政治意识，做到对党忠诚。牢固树立“四个意识”，不折不扣贯彻执行党中央和省委、省政府的决策部署。始终把政府工作置于市委领导之下，真正做到市委提倡的坚决响应、决定的坚决执行、禁止的坚决不做，自觉维护风清气正的政治生态。二是增强服务意识，做到执政为民。牢固树立以人民为中心的发展思想，把增进人民福祉作为发展的出发点和落脚点。特别是困难时期，坚持把有限的资金用在刀刃上，使广大群众的生活得到逐步改善。树立诚信形象，做出的承诺必须及时兑现，出台的政策必须言出法随，提升政府公信力。三是增强时代意识，做到勤于学习。坚决摒弃老思维、老套路、老办法。加强学习实践，建设一支具有专业思维、专业素养和专业方法的干部队伍，不断提高政府工作专业化水平。四是增强规则意识，做到依法行政。严格遵守宪法和法律，运用法治思维和法治方式开展工作，自觉接受市人大及其常委会法律监督和工作监督，主动接受市政协民主监督，广泛接受社会各界和新闻舆论监督，不断提高政府工作法治化水平。五是增强责任意识，做到担当实干。完成全年的工作目标，任务十分艰巨，必须以百倍的勇气挑起重担，必须全力以赴认真实干，必须大胆创新以求突破，打造“鹤岗速度”，推动各项工作落到实处。六是增强纪律意识，做到清正廉洁。认真履行“一岗双责”，严格执行《准则》和《条例》，严肃查处各类违纪违规行为。坚持用制度管权管事管人，加强对公共资金、公共资源、国有资产的监管。延伸经济责任审计领域，强化对重点领域、关键环节、重要岗位权力运行的监督制约。严格执行中央和省、市委廉洁从政的有关规定，节约政府开支，降低行政成本。

各位代表！发展的蓝图已经绘就，奋斗的目标已经明确。让我们更加紧密地团结在以习近平同志为核心的党中央周围，在省委、省政府和市委的正确领导下，按照市第十二次党代会的战略部署，紧紧依靠全市人民，万众一心、奋发图强、苦干实干，为加速推进城市转型，同步建成小康社会，建设活力边城、幸福家园而努力奋斗，以优异成绩迎接党的十九大和省第十二次党代会胜利召开！
